# ChemSex: A Narrative Review of a New Challenge in Addiction Treatment

**DOI:** 10.1192/j.eurpsy.2026.10885

**Published:** 2026-07-31

**Authors:** A. Diehl, A. C. Trevelin, R. A. Bosso, A. Almeida, A. Granjeiro

**Affiliations:** 1 researcher, Abead, Londrina; 2 researcher, Abead, Itapira; 3 researcher, Abead, Campinas, Brazil

## Abstract

**Introduction:**

ChemSex refers to the intentional use of psychoactive substances for sexual purposes, aiming to intensify pleasure, reduce inhibitions, and prolong sexual encounters—particularly among men who have sex with men (MSM). Although the phenomenon has gained visibility in European countries, it remains underexplored in Brazil and other parts of Latin America.

**Objectives:**

This narrative literature review aimed to investigate ChemSex within the Brazilian context and analyze theoretical frameworks that help explain its complexity.

**Methods:**

The study adopted a qualitative and descriptive approach, with searches conducted in national and international databases between 2000 and 2025. Empirical and theoretical studies focusing on ChemSex among MSM were included, especially those relevant to the Brazilian context. Thematic analysis was guided by theoretical references from the sociology of sexuality, social psychology, and studies on drugs and public health.

**Results:**

Findings indicate that ChemSex is linked to multiple dimensions: physical and mental health, public policy, social vulnerabilities, and community dynamics. Addressing the phenomenon requires intersectoral responses that include individuals engaged in the practice, promoting person-centered approaches and collaboration between health services, public safety, and community organizations. Despite some progress, structural gaps persist, such as institutional stigmatization and the lack of adequate services for vulnerable populations—including migrants, ethnic minorities, sex workers, and trans people.

The theoretical analysis revealed ChemSex as a multifaceted practice. The Identity Process Theory highlights substance use as a coping strategy in response to threats to self-esteem and belonging. Syndemic Theory emphasizes the interaction of social and health factors that amplify vulnerabilities. Sexual Performance Theory interprets sex as a performative act shaped by cultural and technological norms. Queer Theory views ChemSex as a dissident practice that challenges heteronormative standards. Lastly, Gay Community Stress Theory points to internal pressures within the LGBTQIAPN+ community that contribute to psychological distress and engagement in ChemSex.

**Conclusions:**

ChemSex cannot be understood solely through a biomedical or psychosexual lens. It is essential to incorporate social, cultural, and political dimensions that shape experiences of pleasure, risk, and exclusion. Interventions should promote education around empowerment, consent, and self-determination, acknowledging the complexity of the phenomenon and the urgency of integrated, sensitive responses tailored to the realities of LGBTQIAPN+ populations.

**Disclosure of Interest:**

None Declared

